# Sex Differences in Type 2 Diabetes Mellitus-Related Left Ventricular Remodeling: A Cardiovascular Magnetic Resonance Study

**DOI:** 10.1155/2022/1427864

**Published:** 2022-05-25

**Authors:** Yongning Shang, Yulin Zhang, Weiling Leng, Xiaotian Lei, Liu Chen, Xiaoyue Zhou, Ziwen Liang, Jian Wang

**Affiliations:** ^1^Department of Ultrasound, Southwest Hospital, Army Medical University (Third Military Medical University), Chongqing, China; ^2^Department of Radiology, Southwest Hospital, Army Medical University (Third Military Medical University), Chongqing, China; ^3^Department of Cardiology, Xinqiao Hospital, Army Medical University (Third Military Medical University), Chongqing, China; ^4^Department of Endocrinology, Southwest Hospital, Army Medical University (Third Military Medical University), Chongqing, China; ^5^Siemens Healthineers Ltd, Shanghai, China

## Abstract

**Background:**

The purpose of this study was to evaluate the sex differences in myocardial structure, tissue characteristics, and myocardial function in type 2 diabetes mellitus (T2DM) patients.

**Methods:**

A total of 62 T2DM patients and 40 controls were prospectively recruited for the study. All the participants were scanned using cardiovascular magnetic resonance (CMR) cine and underwent native and postcontrast T1 mapping to obtain left ventricular (LV) structure, function, and tissue characteristics. The differences between the control and T2DM patients were compared in males and females, respectively.

**Results:**

For myocardial structure, T2DM was associated with a larger ratio of myocardial mass to end-diastolic volume (MVR, T2DM: 0.87 ± 0.20 vs. controls: 0.73 ± 0.14, *p* = 0.008) and thicker wall thickness (WT, T2DM: 6.5 ± 1.1 mm vs. controls: 5.6 ± 1.0 mm, *p* = 0.002) in females. For tissue characteristics, T2DM was associated with a similar T1 value, elevated extracellular volume fraction (ECV, T2DM: 27.8 ± 3.6% vs. controls: 25.1 ± 2.5%, *p* = 0.002), and increased extracellular matrix volume index (ECMVi, T2DM: 15.8 ± 3.8 ml/m^2^ vs. controls: 13.4 ± 2.7 ml/m^2^, *p* = 0.008) in males. For myocardial function, in male, compared with control, T2DM was associated with decreased peak longitudinal diastolic strain rate (PLDSR, T2DM: 0.97 ± 0.19 1/s vs. control: 1.13 ± 0.29 1/s, *p* = 0.030).

**Conclusions:**

There might be sex differences in myocardial remodeling induced by T2DM, including LV structural concentric remodeling in female patients and extracellular matrix remodeling and subclinical diastolic dysfunction in male patients.

## 1. Introduction

Type 2 diabetes mellitus (T2DM) is one of the most common chronic diseases affecting health globally [1]. T2DM patients are at increased risk for heart failure (HF) [2]. Studies have shown that female patients have a higher risk of HF than males. The type of HF (heart failure with preserved or reduced ejection fraction) also differs between male and female patients [3], indicating that there could be a sex difference in T2DM-related myocardial changes.

Previous studies indicate that T2DM patients are associated with diffuse myocardial extracellular interstitial fibrosis [4], structural concentric remodeling [5] and functional alterations [6], including diastolic dysfunction, and finally at increased risk for HF. However, currently there is a lack of comprehensive research about sex difference in T2DM-related myocardial extracellular matrix, structural and functional alterations.

Cardiovascular magnetic resonance (CMR) has been widely used to comprehensively evaluate tissue characteristics and the structure and function of the myocardium due to its advantages of high spatial resolution, good repeatability, a large field of vision, and multiparameter imaging. T1 mapping and the extracellular volume fraction (ECV) can be used to quantify the extracellular matrix of the myocardium [7]. CMR cine is considered the “gold standard” for measuring the structure and function of the myocardium [8, 9].

Therefore, we used CMR techniques, including cine and T1 mapping, to evaluate sex differences in myocardial structure, tissue characteristics, and myocardial function in patients with T2DM vs. controls.

## 2. Materials and Methods

### 2.1. Study Patients

This study was approved by the Ethics Committee of First Affiliated Hospital of Third Military Medical University (number: 2016 scientific research No. 50, approval time: May 11, 2016), and all subjects given written informed consent. The study prospectively enrolled T2DM patients. The inclusion criteria were as follows: (1) an initial diagnosis of T2DM, according to World Health Organization criteria [10] and (2) no history of cardiovascular disease or chest pain. The exclusion criteria were as follows: (1) hypertension (resting systolic/diastolic blood pressure > 140/90 mmHg), (2) glomerular filtration rate ≤ 30 mL/min/1.7 m^2^, and (3) other CMR contraindications. Controls were enrolled from the community with the following criteria: (1) no T2DM, (2) no hypertension, and (3) no history of cardiovascular disease or chest pain. In total, 62 T2DM patients and 40 controls were recruited for the study.

### 2.2. Anthropometry and Biochemistry Exams

As previously reported [11], the height, weight, and blood pressure of all the subjects were measured. Venous blood samples were collected half an hour before each CMR examination and sent to the laboratory and nuclear medicine department for the examination of hematocrit (HCT), fasting blood glucose, glycosylated hemoglobin (HbA1c), triglycerides, total cholesterol, high-density lipoprotein (HDL), and low-density lipoprotein (LDL) cholesterol.

### 2.3. CMR Protocols

As previously reported [11], CMR scanning was performed on a 3.0T MR scanner (MAGNETOM Trio, Siemens Healthcare, Erlangen, Germany) with a 12-channel body array coil. A prototype CV shimming for patient-specific shimming of the heart was used to improve field uniformity.

An electrocardiogram-gated, breath-hold, balanced steady-state free-precession (bSSFP) sequence was used to obtain four-chamber long-axis cine images and short-axis cine images covering the entire LV, and the parameters were as follows: slice thickness, 6 mm; gap, 1.5 mm; field of view, 325 × 400 mm^2^; matrix, 179 × 256; TR/TE, 59.22/1.45 ms; and 25 reconstructed phases per cardiac cycle.

An electrocardiogram-gated, breath-hold, MOdified Look-Locker Inversion recovery (MOLLI) prototype sequence was performed with the following parameters: a bSSFP readout; field of view, 400 × 300 mm^2^; matrix, 256 × 166; TR/TE, 301.7/1.09 ms; flip angle, 35 degrees; and 6 mm thickness. Midventricular short-axis images were acquired before and approximately 15 min after the administration of 0.2 mmol/kg gadoteric acid meglumine (Dotarem, Guerbet, BP7400, F95943, Roissy CdG Cedex, France) with a 5(3)3 and 4(1)3(1)2 sampling pattern for native and postcontrast T1 mapping, respectively. T1 maps were generated inline from the MOLLI images with motion correction (MOCO).

### 2.4. Data Acquisition

All the cine and T1 map images were analyzed offline using the cvi42 software (Circle Cardiovascular Imaging Inc., Calgary, Alberta, Canada). Endo- and epicardial contours on the cine images were manually traced to obtain the LV end-diastolic volume index (EDVi), end-systolic volume index (ESVi), stroke volume index (SVi), ejection fraction (EF), cardiac index (CI), myocardial mass index (MMi) with papillary muscle included in the LV lumen, and the ratio of MMi to EDVi (MVR). The wall thickness (WT) on the LV middle short-axis interventricular septum was measured. Two-dimensional feature tracking was performed on four-chamber long-axis cine images and midventricular short-axis cine images to obtain global longitudinal, circumferential, and radial strain and the peak systolic and diastolic strain rates.

As previously reported [11], an ROI was manually selected on the middle third of the myocardial interventricular septum and another ROI in the LV cavity to obtain myocardial and blood T1 values. The ECV was calculated from native and postcontrast T1 of myocardium and blood and HCT [12]. The extracellular matrix volume index (ECMVi) and cellular volume index (CVi) were calculated according to the myocardial mass index and ECV, using the published formula [13].

### 2.5. Statistical Analysis

The data were analyzed using SPSS (version 21.0, SPSS Inc., Chicago, IL, USA) and GraphPad Prism software (version 6.01, GraphPad Software, Inc., La Jolla, CA, USA). The Kolmogorov–Smirnov test was used to test the normality of the variables. For continuous and normal variables, differences between the means were compared using one-way ANOVA and least significant difference (LSD) test was performed to evaluate post hoc comparisons. The intra- and interobserver variabilities of CMR indices were analyzed by determining the intraclass correlation coefficient (ICC) and mean difference. Statistical tests were two-tailed, and statistical significance was defined as *p* < 0.05.

## 3. Results

### 3.1. Participant Characteristics

The demographic and biochemical data are presented in Table 1. There was no significant difference in age, body surface area, body mass index, or blood pressure between male T2DM and male controls. There was also no significant difference in age, body surface area, body mass index, or blood pressure between female T2DM and female controls. Compared with the female controls, female T2DM was associated with higher glucose and glycated hemoglobin. Compared with the male controls, male T2DM was also associated with higher glucose and glycated hemoglobin.

### 3.2. Sex Differences in Structural, Functional, and Tissue Characteristics in the Control Group

**Figure 1:**
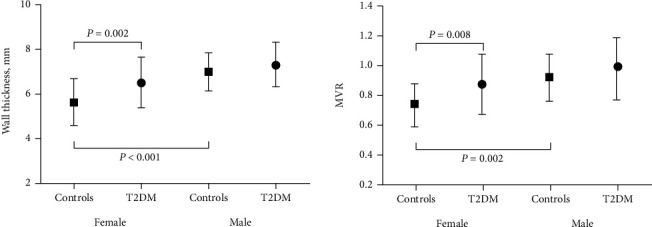
Sex differences in myocardial structure between control and T2DM subjects. MVR: mass to end-diastolic volume ratio.

**Figure 2:**
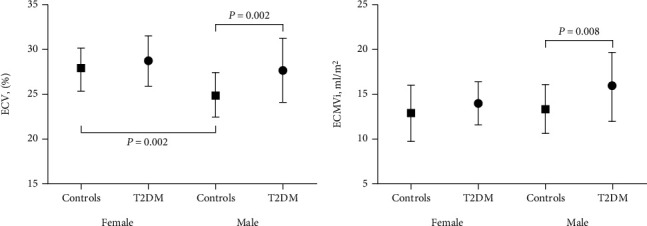
Sex differences in myocardial tissue characteristics between control and T2DM subjects. ECV: extracellular volume fraction; ECMVi: extracellular matrix volume index.

For myocardial structure, female controls had thinner WT (females: 5.6 ± 1.0 mm vs. males: 7.0 ± 0.9 mm, *p* < 0.001) and smaller MVR (females: 0.73 ± 0.14 vs. males: 0.91 ± 0.15, *p* = 0.008, Figure 1). For tissue characteristics, compared with male controls, female controls had larger native T1 values (females: 1240.0 ± 60.0 ms vs. males: 1190.9 ± 44.2 ms, *p* = 0.027) and larger ECV (females: 28.4 ± 2.6% vs. males: 25.1 ± 2.5%, *p* = 0.001, Figure 2). For myocardial function, female controls had a larger radial strain (females: 50.1 ± 9.1% vs. males: 41.3 ± 6.5%, *p* = 0.001), larger circumferential strain (females: −23.2 ± 2.5% vs. males: −21.1 ± 2.3%, *p* = 0.004), larger radial diastolic strain rate (females: −3.5 ± 1.1 1/s vs. males: −2.7 ± 0.7 1/s, *p* = 0.006).

### 3.3. Sex Differences in Structural, Functional, and Tissue Characteristics in the T2DM Group

For myocardial structure, T2DM was associated with smaller EDVi (T2DM: 60.3 ± 8.0 ml/m^2^ vs. controls: 67.3 ± 9.5 ml/m^2^, *p* = 0.008), similar MMi, larger MVR (T2DM: 0.87 ± 0.20 vs. controls: 0.73 ± 0.14, *p* = 0.008), and thicker WT (T2DM: 6.5 ± 1.1 mm vs. controls: 5.6 ± 1.0 mm, *p* = 0.002) in females. In males, there were no significant differences in any of these indices between T2DM patients and controls (Figure 1).

For the tissue characteristics, there were no significant differences in T1 values, ECV, ECMVi, or CVi between T2DM patients and controls in females. But in males, T2DM patients were associated with a similar T1 value, elevated ECV (T2DM: 27.8 ± 3.6% vs. controls: 25.1 ± 2.5%, *p* = 0.002), and increased ECMVi (T2DM: 15.8 ± 3.8 ml/m^2^ vs. controls: 13.4 ± 2.7 ml/m^2^, *p* = 0.008) (Figure 2).

**Figure 3:**
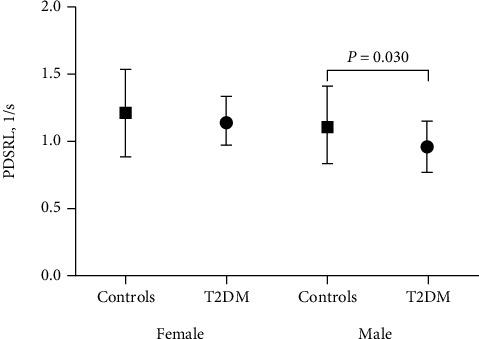
Sex differences in myocardial function between control and T2DM subjects. PDSRL: peak longitudinal diastolic strain rate.

For myocardial function, there were no significant differences in the EF, strain, systolic, or diastolic strain rates between T2DM patients and controls in females. In males, T2DM was associated with decreased peak longitudinal diastolic strain rate (PDSRL, T2DM: 0.97 ± 0.19 1/s vs. controls: 1.13 ± 0.29 1/s, *p* = 0.030) (Figure 3).

## 4. Discussion

In this study, we found that there was LV structural concentric remodeling in female T2DM patients and extracellular matrix remodeling and subclinical diastolic dysfunction in male T2DM patients.

There was no change seen in MMi, but decreased EDVi, increased MVR, and thicker WT were noted in patients with T2DM, indicating LV concentric remodeling, which is consistent with previous studies [5, 11]. LV concentric remodeling could be related to insulin resistance and relative insulin insufficiency in patients with T2DM, leading to a lipid metabolism disorder of the myocardium. The lipid toxicity in the myocardium results in the thickening of myocardial cells and concentric remodeling [5]. Myocardial steatosis has been shown to be modifiable [14, 15], which could improve concentric remodeling. Therefore, early detection of structural remodeling in patients with T2DM will be conducive to timely treatment to prevent its further development and reduce the occurrence of adverse events.

Previous studies have shown myocardial extracellular matrix remodeling in patients with T2DM [4, 16]. This is consistent with the present study, which included increased ECV and ECMVi. The typical pathological change seen in diabetic cardiomyopathy is diffusely increased collagen in the extracellular matrix, which is primarily due to an increase of protein kinase C in fibroblasts caused by T2DM lipotoxicity and hyperglycemia, stimulating them to produce more collagen that is deposited in the extracellular matrix [17]. The increase of extracellular matrix components can increase myocardial stiffness and affect the diastolic function of the myocardium. In the present study, subclinical diastolic dysfunction in T2DM patients could be related to an increase in extracellular matrix components. Increased extracellular matrix components will lead to “vulnerable myocardium” and increase the occurrence of arrhythmias, heart failure, and other adverse events. The results of this study indicated that T1 mapping might be helpful for the early detection of extracellular matrix changes in patients with T2DM and provides more information for early treatment.

This study showed that there are sex differences in the above LV structure, tissue characteristics, and functional changes, including LV concentric remodeling in female T2DM patients and, in contrast, extracellular matrix remodeling and subclinical diastolic dysfunction in male T2DM patients. Previous studies have focused on the effects of sex differences on different myocardial remodeling patterns, including aortic stenosis [18] and transfemoral aortic valve replacement [19]. However, a few studies on sex differences in T2DM can be found in the literature. Athithan et al. [20] showed that there was more severe concentric remodeling in male patients, and left ventricular and left atrial function was decreased, whereas these changes were not found in female patients. However, this study did not focus on the changes in the extracellular matrix. In detail, there was more severe concentric remodeling in male patients in study of Athithan et al. [20]; however, our present study showed that there was more severe concentric remodeling in female patients. Both we and Athithan et al. think that concentric remodeling may be caused by myocardial fat deposition. In our present study, female T2DM patients were associated with worse HDL, which maybe indicated more myocardial fat deposition in female patients [21]. Indeed, 1H-MRS can be used to quantity myocardial fat deposition to determine myocardial lipotoxicity and LV concentric remodeling in female patients. There was subclinical diastolic dysfunction in male T2DM patients, which is consistent with previous study [20]. Taking account that the diastolic dysfunction is the main described characteristic of the diabetic cardiomyopathy, it suggests that most studies are slanted towards male patients. In the future, more research might be needed to explore sex differences in T2DM-related myocardial changes.

The results of the present study provide some reference information for different types of myocardial remodeling and the corresponding treatment strategies in different sex patients with T2DM. Previous studies [16, 22] have shown that renin-angiotensin-aldosterone system (RAAS) inhibitors can alleviate the increase in extracellular matrix components to some extent. Therefore, male patients with early extracellular matrix changes and subclinical diastolic dysfunction might need the intervention of RAAS inhibitors. Female patients with early myocardial concentric remodeling could need more treatment for lipotoxicity caused by myocardial fat deposition.

Our study had several limitations. First, the overall number of subjects was relatively small. Second, we did not recruit patients with a GFR ≤ 30 mL/min/1.7 m^2^, and the patients in the study received different treatments. Third, this study was a cross-sectional one, so there was no follow-up analysis of the above changes in patients with T2DM.

In conclusion, there might be sex differences in myocardial remodeling induced by T2DM, including LV structural concentric remodeling in female patients and extracellular matrix remodeling and subclinical diastolic dysfunction in male patients.

## Figures and Tables

**Table 1:** Demographic and biochemical data of all participants.

	Females		Males		*p* value
	Control (*n* = 21)	T2DM (*n* = 32)	Control (*n* = 19)	T2DM (*n* = 30)	
Age, years	53.0 ± 10.2	55.7 ± 8.0	47.7 ± 13.1^∗∗^	53.5 ± 7.9	0.045
Duration of diabetes, years		7 (4-11)		8 (4-11.3)	
Height, m	1.57 ± 0.04	1.57 ± 0.06	1.68 ± 0.06^∗∗∗^	1.67 ± 0.06	<0.001
Weight, kg	56.1 ± 6.6	59.8 ± 9.3	70.0 ± 7.3^∗∗∗^	69.9 ± 9.8	<0.001
Body surface area, m^2^	1.56 ± 0.1	1.61 ± 0.15	1.81 ± 0.11^∗∗∗^	1.8 ± 0.15	<0.001
Body mass index, kg/m^2^	22.8 ± 2.6	24.2 ± 2.7	24.8 ± 2.4^∗^	25.1 ± 2.9	0.023
Systolic blood pressure, mmHg	115.2 ± 9.2	120.5 ± 9.8	120.5 ± 10.2	119.7 ± 9.7	0.217
Diastolic blood pressure, mmHg	79.2 ± 8.1	78.3 ± 7.7	80.9 ± 5.6	80.0 ± 6.9	0.604
Total cholesterol, mmol/L	5.7 ± 2.1	5.2 ± 1.5	5.0 ± 0.7	5.0 ± 1.7	0.491
Triglycerides, mmol/L	1.4 ± 0.9	2.2 ± 1.7	1.8 ± 0.9	3.1 ± 3.0	0.040
HDL-C, mmol/L	1.6 ± 0.4	1.3 ± 0.3^##^	1.2 ± 0.2^∗∗^	1.1 ± 0.4	<0.001
LDL-C, mmol/L	3.7 ± 1.7	3.2 ± 1.0	3.2 ± 0.5	2.9 ± 1.0	0.185
Glucose, mmol/L	5.3 ± 0.6	8.6 ± 3.6^#^	5.5 ± 0.6	10.6 ± 7.6^&^	0.001
Glycated hemoglobin, %	5.5 ± 0.4	8.1 ± 2.0^###^	5.4 ± 0.5	8.5 ± 2.1^&&&^	<0.001

HDL-C: high-density lipoprotein cholesterol; LDL-C: low-density lipoprotein cholesterol. Comparison between control and T2DM in females, ^#^*p* < 0.05, ^##^*p* < 0.01, and ^###^*p* < 0.001. Comparison between control and T2DM in males, ^&^*p* < 0.05, ^&&^*p* < 0.01, and ^&&&^*p* < 0.001. Comparison between female control and male control, ^∗^*p* < 0.05, ^∗∗^*p* < 0.01, and ^∗∗∗^*p* < 0.001.

## Data Availability

The research data used to support the findings of this study are available from the corresponding author upon request.

## Abbreviations

bSSFP: Balanced steady-state free-precession
CI: Cardiac index
CMR: Cardiovascular magnetic resonance
CVi: Cellular volume index
ECMVi: Extracellular matrix volume index
ECV: Extracellular volume fraction
EDVi: End-diastolic volume index
EF: Ejection fraction
ESVi: End-systolic volume index
HbA1c: Glycosylated hemoglobin
HCT: Hematocrit
HDL: High-density lipoprotein
HF: Heart failure
ICC: Intraclass correlation coefficient
LDL: Low-density lipoprotein
LSD: Least significant difference
LV: Left ventricle
MMi: Myocardial mass index
MOCO: Motion correction
MOLLI: MOdified Look-Locker Inversion recovery
MVR: The ratio of MMi to EDVi
SVi: Stroke volume index
T2DM: Type 2 diabetes mellitus
WT: Wall thickness.
